# Evaluation of a community awareness programme to reduce delays in referrals to early intervention services and enhance early detection of psychosis

**DOI:** 10.1186/s12888-015-0485-y

**Published:** 2015-05-02

**Authors:** Brynmor Lloyd-Evans, Angela Sweeney, Mark Hinton, Nicola Morant, Stephen Pilling, Judy Leibowitz, Helen Killaspy, Sanna Tanskanen, Jonathan Totman, Jessica Armstrong, Sonia Johnson

**Affiliations:** Division of Psychiatry, University College London, Charles Bell House, 67-73 Riding House Street, W1W 7EJ London, United Kingdom; Camden and Islington NHS Foundation Trust, Early Intervention Service, 4 Greenland Road, Camden, NW1 London, United Kingdom; Department of Clinical, Education and Health Psychology, University College London, 1-19 Torrington Place, WC1E 7HB London, United Kingdom; Camden and Islington NHS Foundation Trust, Psychological Therapies Services, St Pancras Hospital, 4St Pancras Way, NW1 0PE London, United Kingdom; Department of Psychology, University of Bath, BA2 7AY, 2SouthBath, United Kingdom

**Keywords:** Psychosis, Early intervention, Health education

## Abstract

**Background:**

Reducing treatment delay and coercive pathways to care are accepted aims for Early Intervention Services (EIS) for people experiencing first episode psychosis but how to achieve this is unclear. A one-year community awareness programme was implemented in a London EIS team, targeting staff in non-health service community organisations. The programme comprised psycho-educational workshops and EIS link workers, and offering direct referral routes to EIS. Its feasibility and its impact on duration of untreated psychosis and pathways to EIS were evaluated.

**Methods:**

Evaluation comprised: pre and post questionnaires with workshop participants assessing knowledge and attitudes to psychosis and mental health services; and a comparison of new service users’ “service DUP”(time from first psychotic symptom to first contact with EIS) and pathways to care in the intervention year and preceding year. Focus groups sought stakeholders’ views regarding the benefits and limitations of the programme and what else might promote help-seeking.

**Results:**

41 workshops at 36 community organisations were attended by 367 staff. 19 follow up workshops were conducted and 16 services were allocated an EIS link worker. Participants’ knowledge and attitudes to psychosis and attitudes to mental health services improved significantly following workshops. In the year of the intervention, only 6 of 110 new service users reached EIS directly via community organisations. For all new referrals accepted by EIS, in the intervention year compared to the previous year, there was no difference in mean or median service DUP. A clear impact on pathways to care could not be discerned. Stakeholders suggested that barriers to referral remained. These included: uncertainty about the signs of early psychosis, disengagement by young people when becoming unwell, and worries about stigma or coercive treatment from mental health services. More general, youth focused, mental health services were proposed.

**Conclusions:**

The community awareness programme did not reduce treatment delays for people experiencing first episode psychosis. Further research is needed regarding effective means to reduce duration of untreated psychosis. Although EIS services are guided to promote access through community engagement, this may not be an effective use of their limited resources.

**Trial registration:**

Current Controlled Trial ISRCTN98260910 Registered 19th May 2010.

**Electronic supplementary material:**

The online version of this article (doi:10.1186/s12888-015-0485-y) contains supplementary material, which is available to authorized users.

## Background

Long duration of psychosis (DUP) is associated with poor outcomes for people experiencing a first episode of psychosis [1,2] and poorer quality of life at first contact with services [1]. However, long DUP is common: with mean DUP of up to two years [1], and median DUP of six months or more reported [3]. Early Intervention Services (EIS), which seek to provide intensive, specialist support to young people with a first episode psychosis, have been set up internationally and were mandated nationally in England in 2000 [4]. EIS teams have a specific aim of minimising DUP [5], in order to provide effective treatment during the early “critical period” following onset of psychosis [6], to improve long-term outcomes [7]. However, establishing an EIS team may, by itself, not reduce DUP [8]. Recent UK studies in the context of established EIS services, have still found over a third of people with first episode psychosis with a DUP of over six months [9,10], and high proportions of service users with aversive pathways to care, including over a third via criminal justice services for Black minority ethnic groups; over half via emergency health services for most groups) [11].

Finding means to reduce treatment delays and avoid coercive pathways to care for people with first episode psychosis is therefore of high importance. Contributory factors to DUP include: lack of trust in mental health services and fear of coercion; fear of stigma and discrimination; failing to recognise problems as psychosis; not knowing where or how to access support; and unhelpful service responses [12-15]. DUP comprises several components: delays at any stage (e.g. initial help-seeking, in primary care, or within secondary mental health services) may be responsible for long DUP overall [9]. About half of DUP may typically constitute delays in initial help-seeking, before any contact with health services is made [16,17]. A systematic review of initiatives to reduce DUP [18] found that only large public awareness campaigns had in some instances been successful in reducing DUP, and that education campaigns targeting GPs had been unsuccessful [17,19]. The review concluded that initiatives to reduce DUP need to encourage initial help-seeking as well as subsequent swift health service response. It highlighted the lack of evidence about initiatives directed at workers from non-health community organisations(such as teachers, school counsellors, youth workers, housing or employment service staff, or leaders of faith groups or community organisations), who might already be supporting young people at the point of onset of psychosis, and therefore potentially able to encourage help-seeking.

The role of workers in non-health community organisations in pathways to EIS care for people with FEP is not well understood and may vary in different social/cultural contexts. A Canadian study found that non-health community workers are commonly involved in pathways to EIS care, and that their involvement is associated with longer DUP [20]; a British study by contrast, found an association between involvement of non-health community workers and shorter DUP [21]. A recent qualitative study of experiences of help-seeking for a first episode of psychosis in the UK [15] found that community workers were willing to support people to access help, but identified challenges to community workers’ involvement in help-seeking (failure to recognise difficulties, perceptions that “becoming involved” might exceed the boundaries of their role, tendency to adopt a “wait and see” approach).. The response of non-health community workers to someone experiencing the onset of psychosis may therefore be amenable to change. Because these community workers may have existing relationships of trust with young people who are developing psychosis and be well placed to notice changes of mood and behaviour, they constitute a promising target for initiatives to improve pathways to appropriate care and to reduce treatment delays.

### Aims

This study used mixed methods to investigate the feasibility and acceptability of an Early Detection (ED) programme in the catchment area of an inner London EIS team, and to provide a preliminary evaluation of its effectiveness in improving access and pathways to an EIS service. The ED programme targeted staff working in non-health community organisations, who might be in contact with and be supporting young people at the start of a first episode of psychosis. The primary aims of the ED programme were to encourage referrals of people experiencing a possible first episode psychosis directly to the EIS team from community organisations, thus reducing service DUP (i.e. time from first psychotic symptom to first contact with EIS) and the number of steps in pathways to EIS care. The objectives of the research study were:

- to develop an intervention to enhance early detection of psychosis and describe its implementation and participants;
- b)to evaluate the impact of ED psycho-educational workshops on community workers’ knowledge of and attitudes towards psychosis and mental health services, and their willingness to refer people to mental health services;
- to compare service DUP and pathways to care for people experiencing a first episode of psychosis referred to the Camden and Islington Early Intervention Service (CIEIS) in the year of the intervention and the preceding year, hypothesising that people referred in the year of the ED programme would have shorter service DUP, and fewer steps and less contact with other mental health services in their pathways to CIEIS care.
- to compare the characteristics, health status, social functioning and experience of care of people referred to CIEIS via routes attributable to the EDprogramme (i.e. self-referrals and referrals direct from non-health organisations) versus those referred via other routes during the year of the initiative, hypothesising that those referred through ED routes will have better outcomes at first contact with CIEIS.
- to explore stakeholders’ views of the ED initiative and the reasons for its success or lack of effectiveness

## Methods

### 1. The study intervention

The ED programme targeted staff working with young people in non-health community organisations in the London Boroughs of Camden and Islington. The programme ran for one year from May 2009 – May 2010 and had five components:

1. A half-day workshop designed to increase participants’ awareness of symptoms of early psychosis and willingness to refer to CIEIS was delivered by CIEIS staff to local community workers in their workplaces.
2. A CIEIS link worker offered monthly meetings and a consistent point of contact for each community organisation which participated in a workshop, aiming to help community staff identify young people potentially experiencing early psychosis and to address barriers to referrals to CIEIS.
3. A one hour top-up training session was offered to each organisation 6–9 months after the initial workshop, designed to reinforce knowledge about signs and symptoms of early psychosis and further encourage referrals to CIEIS.
4. Educational and promotional materials briefly summarising signs of potential early psychosis and encouraging contact with CIEIS were distributed to community organisations electronically and in hard copy. The CIEIS service website was revised to reinforce these messages prominently.
5. CIEIS referral processes were altered to accept direct referrals from any source, rather than from health professionals only, as had been the practice in the service. Individuals, family and community workers were also encouraged to ring up for discussion or advice if concerned about someone with possible psychosis, without providing the name of the person being discussed if preferred, if they did not feel ready to make a referral at that point.

The ED workshops were designed following comprehensive development work including: a systematic review of initiatives to reduce DUP [18]; self-report questionnaire data from staff in community organisations who work with young people about their experiences, attitudes and knowledge of psychosis [22]; interviews with 21 CIEIS users and 9 family members about their experiences of early psychosis and help-seeking [15]; and focus groups with staff from local community and educational organisations working with young people and CIEIS staff [23]. The workshops aimed to increase participants’ knowledge and understanding of psychosis, the mental health system and CIEIS; decrease stigma and discrimination against people experiencing psychosis; explore common reservations about referring someone to mental health services and encourage direct referral or informal contact with CIEIS in the event of concerns about a young person with potential early psychosis. Workshops typically lasted three hours and were co-facilitated by CIEIS staff and a study researcher. Workshops involved a PowerPoint presentation, video footage of clinicians, service users and families discussing psychosis and outlined the case for seeking help at the earliest possible point following onset of psychosis. They included audio-taped testimony from CIEIS service users about the service provided, and time for questions and answers and group discussion, in which concerns or questions relevant to staff in the host organisation were aired.

Study researchers identified potential workshop host organisations which worked with young people, through consultation with CIEIS staff and via directories of local services and faith and community groups provided by local councils and voluntary groups. Researchers then contacted the manager of organisations directly by phone or email to ask about willingness to participate in a workshop. Managers contacted in these ways were also asked to identify other potential organisations to participate in the programme. Following a workshop, a CIEIS clinician contacted the manager of each host organisation to introduce themselves as a contact person for CIEIS and to offer monthly meetings to discuss concerns or potential referrals. Between six and nine months after the initial ED workshop, a study researcher contacted each organisation and offered a one hour follow-up workshop. These were also co-facilitated by a CIEIS clinician and a study researcher.

### 2. Evaluation of the intervention

Evaluation of the early detection programme comprised 4 parts:

- Questionnaires with workshop participants about knowledge and attitudes to psychosis and mental health services before and after the workshop
- A comparison of anonymised, routinely collected service data for service DUP and pathways to care for all new referrals accepted by CIEIS in the year of the early detection programme and the preceding year, to test the hypotheses that, in the year of the study intervention, mean service DUP was shorter and pathways to CIEIS were significantly different from the previous year (involving less contact with mental health services and criminal justice agencies).
- Comparison of the characteristics and experience of contact with mental health services of new referrals to CIEIS via non-health “early detection” routes (self-referrals or referrals from workers in non-health service community organisations) with those referred to CIEIS via health services in the year of the early detection programme, collected by a research interview with consenting participants
- Qualitative focus groups with stakeholder groups about their experience of the early detection initiative, which were conducted at the end of the year-long early detection programme.

The methods for each part of the evaluation are described below.

### i) Setting and sample

- ED workshops were offered to non-health service organisations with a role in supporting young adults, including identified youth and faith groups (including all identified churches, mosques and synagogues within the CIEIS catchment area), employment, education and housing organisations, Black and minority ethnic community groups, probation and social services, and the police. All staff who worked directly with adolescents/young adults were invited to attend.
- Routine service data were collected for all new service users accepted for treatment by CIEIS in the year of the early detection intervention (May 2009 – April 2010) and the preceding year. In a service development unconnected to the study, the catchment area for CIEIS expanded early in the study intervention year to include the whole of the London boroughs of Camden and Islington; previously, CIEIS had only served some sectors of each borough. Data for all referrals to CIEIS were collected notwithstanding this change.
- All new service users accepted for treatment by CIEIS in the year of the early detection programme who met the study inclusion criteria (assessed as having capacity to consent and the ability to understand English) were invited to participate in a research interview as soon as possible following acceptance by the CIEIS.
- Focus groups with 6–10 participants were convened, one group with each of the following stakeholder groups: (a) front-line staff from the community organisations in the ED intervention; (b) managers in these organisations; (c) CIEIS staff; (d) CIEIS service users; and (e) their families. Service user and family member participants were purposively sampled to include a range of ages, ethnic groups and referral routes, whilst community organisation staff represented a range of types of organisation and levels of seniority.

### ii) Measures

- **Workshop Questionnaires** for use before and after the ED workshops were adapted from questionnaires about stigma relating to psychosis developed by Angermeyer and Matschinger [24-26] and a UK survey of knowledge about psychosis among staff in educational services [22]. These assessed: i) *Knowledge about psychosis*: participants rated whether 17 statements (which included common symptoms and red herrings) suggested possible psychosis or not, creating a total score ranging from 0–17; ii) *Attitude to psychosis*: participants rated level of agreement with eight stigmatising statements about people with psychosis on a five point Likert scale, creating a total score from 8–40; iii) *Attitude to mental health services*: participants rated agreement with 12 positive and (reverse scored) negative statements about mental health services for people with psychosis on a five point Likert scale, creating a total score from 12–60; iv) *Expected outcome for someone following a first episode of psychosis*: participants were asked to choose the most likely from five possible outcomes (ranging from total remission to ongoing deterioration of health) for someone following a first episode of psychosis – first with no treatment; and then with EIS treatment; v) *Willingness to refer to an Early Intervention Service*: participants selected their preferred initial referral destination for someone potentially experiencing a first episode of psychosis from 13 sources, including an Early Intervention Service.
- **Routine data** were originally collected by CIEIS staff using Midata: a system for collecting standard outcomes data, used by EIS teams across London [27]. Anonymised data were retrieved for all new service users accepted for treatment by CIEIS in the year of the study intervention and the preceding year regarding: demographic characteristics, service DUP; and pathways to CIEIS care. DUP data were collected using the Nottingham Onset Schedule [28]: rather than the more usual definition of DUP (time from first psychotic symptom to start of adherence to antipsychotic medication), a measure of “service DUP” more directly relevant to the aims of the ED Programme was used, i.e. the duration from first psychotic symptom to first contact with CIEIS. Pathways to care data were collected using the measure developed for MiData [27]. The number of steps in the referral pathway was recorded. Pathways to CIEIS were categorised as: i) no contact with other mental health services; ii) contact with community mental health services but no acute services (inpatient hospital wards or crisis home treatment teams); or iii) crisis or coercive route - contact with acute mental health services or the police or courts.
- **A research interview** with consenting service users accepted for treatment by CIEIS during the year of the ED programme assessed: demographic characteristics, education and employment status at time of referral to CIEIS, satisfaction with mental health services [29], perception of coercion [30], positive symptoms of psychosis [31], self-harm and violence (using items from the Brief Psychiatric Rating Scale [32], hope and anticipated stigma, social network size [33], and pre-morbid adjustment [34]. A diagnosis for each participant was then generated by researchers using the OPCRIT programme [35]. DUP [28] and pathways to care [27] were also assessed in the research interview (separate to the routine data on DUP and care pathways used in the pre-post evaluation of the ED programme). Full details of measures used in this interview are provided in the data supplement accompanying this paper (Additional file 1: Appendix 1).
- **Focus groups** were conducted by two researchers. Preliminary outcomes from the ED Programme were summarised for participants, then a semi-structured group interview was conducted using topic guides which covered: the usefulness of the ED programme; ways it could have been improved; and its impact on both the referring service and CIEIS.

### iii) Procedures

The study had ethical approval from the London North West Research Ethics Committee (ref 08/HO722/110). Staff participants in ED workshop questionnaires, service users participating in a research interview, and all focus group participants gave written informed consent to take part in the study. The comparison of service users’ characteristics in the year of the ED programme and the preceding year was approved by the London North West Research Ethics Committee as a service evaluation: routinely collected data were retrieved in anonymised form for all service users without their individual consent. Routine data regarding DUP and pathways to care were initially recorded for each service user by their CIEIS key worker. In line with guidance for using the Nottingham Onset Schedule [28], clinicians’ identified the date of service users’ first psychotic symptom through multiple sources including interview with the service user, and wherever possible, consultation with involved family and reports from other health professionals. Clinicians were supported in identifying and recording DUP data in the intervention year and the preceding year by a research worker based in the service, attached to the MIData project [27]. All quantitative data were entered into SPSS for Windows software for data analysis. Qualitative focus groups were audio-recorded, transcribed and entered into QSR Nvivo7 software for data analysis.

### iv) Analysis

Data from all workshop participants who completed pre and post-workshop questionnaires were compared as paired samples. The characteristics, service DUP and pathways to care of CIEIS service users in the year of the ED programme and the preceding year were compared using bivariate tests. Bivariate comparisons of service users referred via an “early detection” (non-health service) route and via other routes were planned. Focus groups were analysed using thematic analysis [36] using a collaborative coding process where an initial coding frame developed by AS was refined following iterative rounds of discussion with the study tem (NM, SJ, BLE) and further coding, allowing the inclusion of inductive, data-driven themes [37].

## Results

### Implementation of the intervention

Forty-one ED workshops were delivered, hosted by 36 different community organisations. A total of 367 staff attended: their characteristics are reported in Table 1. Workshops were attended by a range of 1 to 23 participants (mean = 9 participants). Participants worked most commonly in housing services (29%), Children and Families Social Services or Probation Services (24%) or youth organisations (20%). There were also participants from cultural organisations, employment support services, faith groups, the police and counselling services. There were more women (62%) than men, and the majority of participants were aged 25–44 (65%). Around half of the participants were from a white British, Irish or other background (53%), with over 40% from a minority ethnic or mixed ethnic group. Additionally, two briefer, one-hour workshops were delivered at two sites of a local further education college. These two workshops were attended by about 150 education staff, but no questionnaire data were collected due to time pressures.

**Table 1:** **Characteristics of ed workshop participants**

**Characteristic**		**n (%)**
Type of organisation	Housing services	106 (29%)
	Social services or probation	87 (24%)
	Youth services	73 (20%)
	NHS mental health workers	30 (8%)
	Cultural groups/BME organisations	26 (7%)
	Police	16 (4%)
	Counsellors	15 (4%)
	Further education colleges	7 (2%)
	Employment agencies	4 (2%)
	Faith groups	3 (1%)
	Total	367
Gender	Female	228 (62%)
	Male	132 (36%)
	Missing	7 (2%)
Age group	16-24	25 (7%)
	25-34	136 (37%)
	35-44	104 (28%)
	45-54	63 (17%)
	54+	20 (5%)
	Missing	19 (5%)
Ethnicity	White British	136 (37%)
	White Irish	23 (6%)
	White other	36 (10%)
	Black Caribbean	50 (14%)
	Black African	36 (10%)
	Black other	5 (1%)
	Asian (Indian, Pakistani, Bangladeshi)	24 (7%)
	Chinese	9 (3%)
	Mixed heritage	9 (3%)
	Other	20 (5%)
	Missing	19 (5%)

All participating organisations were offered a CIEIS link worker and one-hour follow-up training. Nineteen follow-up workshops were delivered, involving staff from 22 community organisations. A total of 178 staff attended the follow-up training: 127 (71%) of participants at follow-up training came from housing services, social services or probation services, or youth groups. Only 46 (26%) of staff who attended the follow-up workshops had attended the initial workshops. Sixteen organisations (44% of the 36 involved in initial workshops) were visited by a CIEIS link worker during the course of the year. The remaining 20 services declined further visits from a CIEIS worker, most commonly citing time pressures and reluctance to prioritise further staff time to discussion of supporting people with potential psychosis.

Leaflets with contact details for CIEIS and information about common signs of early psychosis were distributed at workshops and to GP surgeries locally. Referral processes to CIEIS were changed at the beginning of the ED Programme to allow direct referral to CIEIS from any source, (including self-referral or referral on behalf of another, in writing, or by phone or email or in person, with no referral forms or paperwork preconditions). These more direct referral routes were maintained throughout the year of the programme and advertised prominently on the service website.

### Evaluation of the ED workshops

Of the 367 staff who attended the workshops, 316 (86%) completed pre and post-workshop questionnaires. The workshops were positively appraised: over 97% of staff agreed with statements that the workshops provided useful information about recognising psychosis, that they would recommend contacting CIEIS to a young person with whom they worked and were concerned could be developing psychosis, and that they would ring CIEIS for a confidential discussion and consider referring a young person they supported. The workshops appeared to reach a relevant group of participants: 167 participants (59.4% of those who answered this question) believed they had worked with a young person experiencing psychosis within the last year.

Comparison of paired pre and post-workshop questionnaires found that participants reported modest but significant positive changes to their knowledge about psychosis (mean score rose from 11 to 13 p < 0.001, in a measure with a total range of 0–17), stigmatising attitudes to people with psychosis (mean score fell from 20.2 to 18.7 p < 0.001, in a measure with a total range of 8–40) and attitudes to mental health services (mean score rose from 43.6 to 47.5 p < 0.001, in a measure with a total range of 12–60). The number of people prioritising an EIS service as their first referral destination for someone possibly experiencing early psychosis rose significantly from 37% to 68% (p < 0.001). Additional file 1: Table S1 in the data supplement provides full results. Participants’ expected outcome for someone with psychosis receiving no treatment did not change pre and post-workshop, but significantly more people expected recovery for someone receiving EIS care post workshop - 84% compared to 68% pre-workshop - (see Additional file 1: Table S2 in the data supplement).

### Comparison of new CIEIS service users in the year or the ED programme and the preceding year

In the year preceding the ED programme, 70 new referrals of people experiencing a first episode of psychosis were accepted for treatment by CIEIS. In the year of the ED programme, 110 new referrals were accepted by CIEIS for treatment. However, these figures are not directly comparable, due to the expansion of the CIEIS catchment area during the year of the ED programme. Six of the 110 new referrals (5%) accepted for CIEIS during the year of the intervention may have been attributable to the ED programme. Two came from statutory social services (a Children and Families team and a Youth Offending team); one came from a tutor at a further education college; one from a worker at a local youth group; one from a voluntary sector housing organisation; and one referral came directly from the family of a young person with psychosis, who had seen the contact details on the CIEIS website. These six referrals assessed as people experiencing a first episode of psychosis and therefore eligible for CIEIS treatment constituted 18% of 33 referrals in total to CIEIS from non-health service sources during the study intervention year. This compares with 104 out of 206 referrals (50%) from health services which were accepted by CIEIS for treatment.

Table 2 presents the characteristics and routes to care of service users accepted by CIEIS for treatment in the intervention year and the preceding year. There were no significant differences between the two groups in demographic characteristics or in mean or median service DUP. New CIEIS clients in the year of the ED programme had significantly more steps in their pathway to care, and there were significant differences between the two groups in referral sources, with 17% of new referrals in the ED programme year reaching CIEIS via no contact with other mental health services, compared to 3% in the preceding year. Thirteen of 19 service users who reached CIEIS with no other mental health service contact in the intervention year did so via GP referral rather than via non-health organisations. The proportion of service users reaching CIEIS via crisis or coercive pathways was almost unchanged in the year of the ED programme (64% versus 63% the previous year).

**Table 2:** **Comparison of new referrals to CIEIS in the ED programme year and the previous year**

**Comparison**		**Previous year**	**ED programme year**	**Statistical test**
Number of new referrals accepted for CIEIS treatment		70	110	Not applicable
Proportion of new referrals to CIEIS accepted for treatment		70/123	110/239	Chi^2^ = 3.85
		(57%)	(46%)	p = 0.050
Gender (% male)		50 (71%)	74 (67%)	Chi^2^ = 0.345
(n = 180)				p = 0.557
Age (mean age in years (sd))		24.4 (5.9)	24.3 (6.5)	t = 0.143
(n = 180)				(df = 157.7)
				p = 0.886
Ethnicity	White British	22 (31%)	41 (37%)	Chi^2^ = 4.05
	White Other	8 (11%)	19 (17%)	(df = 5)
	Black ethnic groups	23 (33%)	29 (26%)	p = 0.54
	Asian ethnic groups	10 (14%)	14 (13%)	
	Mixed and other ethnic groups	2 (3%)	4 (4%)	
	Missing	5 (7%)	3 (3%)	
Mean Service DUP - days (sd)		295 (468)	396 (743)	Mann Whitney U test
		n = 66	n = 104	p = 0.715
Median Service DUP - days		133.5	116.5	Independent samples median test
		n = 66	n = 104	p = 0.875
Number of steps in CIEIS pathway to care (mean (sd))		2.06 (0.56)	2.45 (1.08)	t = -3.83 (df =169)
		n = 70	n = 108	[CI -0.64, -0.15]
				p = 0.002
Pathway to CIEIS	No mental health service contact	2 (3%)	19 (17%)	Chi^2^ = 11.57
	Mental health pathway: non-acute	24 (34%)	21 (19%)	p = 0.003
	Mental health acute pathway(acute mental health services or police involved)	44 (63%)	70 (64%)	

### Pathways to EIS in the year of the intervention

Interviews were completed with 63 consenting participants out of the 110 service users accepted for treatment during the year of the ED programme, but these 63 included only two of the six people who accessed the service via early detection routes. Comparisons of the characteristics of early detection service users versus others were therefore not possible. Descriptive data are provided in Additional file 1: Table S3 in the data supplement.

### Stakeholder’s views of the ED intervention

Five focus groups, one with each stakeholder group, were conducted, involving a total of 34 participants (CIEIS service users n = 7; CIEIS carers n = 5; community organisation managers n = 8; community organisation staff n = 7; CIEIS staff n = 7). Focus groups were held in the final two months of the year of the ED programme. Themes derived from analysis of these groups are organised around three main research questions: what was valued in the ED programme; what were the impediments to its success; and what else can we try? Additional quotations from participant interviews illustrating these themes are provided in Additional file 1: Appendix 2 in the data supplement.

### i) What was valued about the ED initiative?

Early detection was seen as important by all participant groups. Community staff and managers described positive contacts with CIEIS such as speed and ease of access and referrals. Participants from these two groups described a number of benefits from participating in the workshops including feeling reassured that the service exists; gaining the understanding, tools and confidence to recognise psychosis; being able to seek advice before a crisis is reached; finding the correct support for people who need it; and developing a language to talk about psychosis.

“And I think they’ve found it very reassuring that they can actually get in touch and have that informal discussion first of all to sort of then see if the referral is then needed”. Community staff

“I think in terms of broadening our understanding it was a fantastic opportunity”. Community staff

### ii) What were the impediments to the success of the ED initiative?

#### Confusion about the CIEIS and referral processes

A few participants from the community staff group described unhelpful contacts and communication with CIEIS prior to the intervention period. Some confusion surrounding the role and remit of CIEIS and its boundaries with other services remained during the ED programme.

“Where does CAMHS stop and begin and when does the early intervention service stop and begin, it’s all very very hazy“. Community staff

This confusion led one worker to conclude that an initial contact or ‘reception’ service for all youth mental health problems is needed.

#### Community organisation factors

Community workers described not referring people to CIEIS for a number of reasons including not seeing anyone experiencing psychosis; only seeing people who are already in contact with mental health services; not knowing whether a referral is necessary and referring to a different organisation. High staff turnovers in community organisations mean that frequent refreshers are needed.

#### Stigma and fear

Fear of the consequences of making a referral - such as intrusive or coercive responses from mental health services, labelling and the effects of stigma – prevented some people from seeking help, including carers, community organisation staff and church members.

“I was just terrified she’d get sectioned. I was going to do anything to make sure she didn’t get sectioned”. Carer

Indeed, stigma was discussed across all the groups as a major barrier to accessing support.

#### Service user and family factors

The community staff, CIEIS staff and service user groups identified that people experiencing psychosis often become isolated and withdrawn and can feel unable to attend groups, meaning that they are not seen by community organisations. Furthermore, Carers and community staff suggested that service users can hide their difficulties for fear of rejection by friends and family, or may fail to recognise when they are struggling.

“As far as he’s concerned, even now, there’s nothing wrong with him. Everybody else, there’s something wrong with everybody else. He’s fine”. Carer

In some circumstances, families may therefore be better placed than the individual themselves to recognise problems and seek help; however, families and friends also struggled to understand what was happening, often assuming that the problem was simply stress and would pass.

“And most teens, most kids, most youngsters, anyway, they stay in their bedrooms and you wouldn’t even think twice. I mean, I didn’t think twice when he used to stay in his bedroom, and I didn’t think anything of it. I just thought he’s 20 years old”. Carer

#### EIS resistance

CIEIS staff expressed some resistance to their role in the early detection programme. In particular, staff feared being inundated with inappropriate referrals and voiced some reluctance to engaging in 'link-up' work with community organisations due to the time commitment needed and the fear this might deflect them from providing support to service users currently receiving a service from CIEIS.

“We need to make sure we’re giving people when they get to the service the service they need before we start looking for more people for the service. I guess that’s the thing, and if there’s not the resources for that there’s no point just endlessly looking for new people if the… if we haven’t got the way of managing them all.” CIEIS staff

#### Workshop factors

Suggestions for improvements to workshops from community staff and managers included reducing the length, adding more case studies and increasing advertising. As people can forget CIEIS, additional reminders are needed beyond the follow up training sessions.

“There may be something about you all having some visibility within the structures as opposed to just coming once a year, and then disappearing off”. Manager

### iii) What else can we try?

#### Tackling barriers to accessing support

Given the stigma and isolation people experiencing psychosis may face, all groups felt that there is a need to break down barriers so that young people are encouraged to seek support. For example, the name ‘Early Intervention Service’ may be a barrier: community managers, staff and service users suggested some people mayprefer informal drops-ins:

“maybe you should even have like drop-ins where people can come, more or less see what they want, and kind of, you know, check their symptoms and talk a little bit about what they’re going through”. Service user

There widespread agreement among community service staff and managers, carers and service users that mental health education in schools would be helpful, and that public awareness campaigns about psychosis are needed. Campaigns should be national and long-term, and could include documentaries; true life and celebrity stories; soap storylines; multimedia advertisements; and social networking sites. Local strategies should include leaflets and items in local media. One service user felt that campaigns should be targeted at young people in order to change the attitudes of the next generations.

### Who to target

Suggestions from all groups for who to target in future early detection programmes included GPs (as the first port of call); families (as the people who often recognise that something is wrong and may be most highly motivated to seek help); young people; the general public; and the services that people might attend (such as hospital emergency departments and schools).

### Reaching people

Alongside public awareness campaigns, suggestions from community staff and managers, and CIEIS service user and carer focus groups for reaching people included websites targeted at young people, television advertisements aimed at worried parents and an outreach worker who would have a consistent presence in community groups. Throughout the groups there was discussion of the importance of using appropriate and non-medical language in reaching out to young people, with CIEIS having a role in supporting workers’ communication styles.

“Just language as well, you know, talking, as I say, not necessarily always about mental health, but about emotional wellbeing … I think it’s the mental health; it just puts young people on edge straightaway, and you’ve already got so many issues going on often. So I think there’s something there about supporting workers in the language that they use”. Manager

## Discussion

### Main findings

Although ongoing contact between community organisations and CIEIS link workers was less consistent than planned, the Early Detection programme in this study appears to have been otherwise well-implemented, and yet ineffective. Workshops with community organisations were well received and engaged large numbers of the target audience, i.e. community staff working with young people vulnerable to experiencing early psychosis. However, the intervention resulted in very few direct referrals to CIEIS of people with first onset psychosis and made no significant difference to mean or median service duration of untreated psychosis compared to the previous year. During the intervention year, a higher proportion of new referrals reached CIEIS without involvement from other mental health services, but most of these came directly from GPs, who were not the target of the intervention. This finding cannot therefore be confidently attributed to the ED programme. The finding of an increase in steps in the pathway to care during the year of the ED programme is hard to explain: an unintended consequence of the study may have been improved routine recording of initial steps in pathways to care.

Several factors may have contributed to the failure of the intervention. Firstly, the gains in knowledge and changes in attitudes about psychosis and mental health services following the workshops, although significant, were modest, and may have been insufficient to change behaviour. Second, these gains in may not have been sustained throughout the year of the intervention: we were unable to measure participants’ attitudes and knowledge beyond the end of the workshops. Fewer than half the services participating in the initial workshops were engaged actively by a CIEIS link worker during the following year (due to unwillingness to prioritise further time for this from community organisations, and possibly also to a lack of assertive engagement by busy CIEIS clinicians in the absence of a clear, positive initial response from the services they contacted.). Only just over half participated in a second follow-up workshop. Third, high staff turnover in community organisations during the intervention year may have reduced the effectiveness of the workshops: only 26% of those attending follow-up workshops were those who attended the initial sessions. Fourth, although the workshops succeeded in reaching large numbers of relevant community workers, some groups were not reached in large numbers, especially faith groups and some BME communities. Those least well engaged with, or with most reservations about contacting mental health services, are unlikely to have been influenced by this ED programme. Fifth, the qualitative part of this study reveals numerous barriers to community workers referring young people who may be experiencing psychosis to mental health services, which the content of the ED programme may have been unable to fully address. These include practical barriers, relating to ongoing uncertainty about when and where to refer, the limits of their work role, or infrequent contact with the young person concerned. Community workers also expressed concerns about damaging their working relationships with young people or stigmatising them through putting them in contact with mental health services. The medical and institutional language used by mental health services and to an extent retained in the ED programme, may have failed to allay these concerns among community staff, some of whom expressed ecological and social, rather than medical-model understandings of psychosis.

### Strengths and limitations

This study provides a naturalistic evaluation of a well-implemented Early Detection Programme. It addresses an identified gap in current knowledge, regarding the effectiveness of initiatives targeting non-health community staff as a means to improve access to Early Intervention Services for people with first episode psychosis. The study has four main limitations however. First, it is a single site study. CIEIS serves two inner-London boroughs with high levels of deprivation and a young, transient and ethnically very diverse population. Community awareness work may be particularly challenging in these circumstances, and the findings of this study may not be generalizable to other settings. Secondly, it took place over a single year: any cumulative benefits of engagement and ongoing education over time with community organisations could not be identified from this study. Thirdly, the study’s pre-post evaluation does not control for other changes in the local area or mental health service system which may influence access to services. The change in the CIEIS catchment area during the course of the study period provides one obvious potential confounding factor. Fourthly, due to ethical approval constraints and local considerations regarding demarcation of the roles of CIEIS and local Child and Adolescent Mental Health Services, the ED Programme only targeted organisations working with young adults rather than children under 16. So for example, further education colleges participated in workshops, but no schools were contacted. The intervention thus did not promote early detection of psychosis for children and by teachers, which may have limited its impact.

### Implications for policy and practice

One positive conclusion from this study is that providing direct access to an EIS service for the public and any referrer appears to be feasible and not to result in a large burden of inappropriate referrals. Previous studies have highlighted that substantial delays in accessing EIS services can occur following first contact with mental health services [9]. Direct pathways to EIS services are therefore desirable. This study offers some support for EIS services being configured as direct access services.

This study does not suggest however, that an outreach campaign targeting non-health community workers is an effective way to improve access to EIS services and reduce treatment delays. The low incidence of psychosis compared to common mental disorders and the likelihood of people disengaging from community organisations when they become unwell both limit the capacity of community workers to identify and help people with early psychosis to access EIS teams. The high turnover of staff in community organisations demonstrated by the small proportion of original workshop attenders present at follow-up workshops may also limit the impact of the programme. Qualitative feedback from CIEIS staff and staff from other organisations, and the comparatively low take-up of link workers and follow-up training from community organisations, suggest that a more intensive early detection programme for community organisations may not be feasible for EIS teams to provide without additional resources. A more intensive programme might also not be accommodated by community organisations either, whose main focus is not about detecting psychosis or providing mental health support: a shorter programme was suggested by community organisation staff in the qualitative focus group. The negative results from this study complement previous negative findings for GP education campaigns [17,19]. It is unclear how EIS services should undertake effective community awareness work and education: EIS managers might therefore justifiably not see this as a service priority, and it may not be an effective use of teams’ limited resources.

The goals of improving pathways to EIS care and minimising treatment delay remain important, given the high numbers of service users reaching EIS services through coercive routes [11,38], the distress and confusion associated with early psychosis [15] and the association between duration of untreated psychosis and poorer health outcomes [1,2]. This study’s negative findings support the conclusions of a previous literature review [18] that large scale early detection initiatives, involving financial support and planning at a regional or national level, may be required to enhance early detection of psychosis.

### Implications for research

Qualitative findings from this study suggest staff in community organisations may remain reluctant to refer to mental health services, even when they do recognise a young person may be experiencing early psychosis. Staff in non-health community organisations – like their counterparts in health services –may also not sustain engagement with young people when they are first developing psychosis or be sufficiently clear about when someone may be experiencing psychosis to make a referral to EIS services. Initiatives which target young people themselves and their families, who may identify psychosis as a problem requiring health service intervention more promptly than the young person themselves in some instances [15], may therefore be required. These may promote initial help-seeking as well as service response, and may therefore be more promising than further programmes targeting supportive professionals, whether from health or non-health organisations. The growth of social media may provide new ways to provide effective and economical early detection campaigns. The distance afforded those who engage in this medium of contact may better manage stigma concerns and constraints. Further research about affordable and sustainable means to improve access to care for people experiencing a first episode psychosis of is needed. While the ED programme in our study derived from development work with stakeholders, future programmes more informed by theoretical models of how to effect behaviour change (e.g. the COM-B system proposed by Michie and colleagues [39]) also warrant evaluation.

The qualitative evaluation in this study indicated that the stigma associated with psychosis, and reservations about EIS services, remained barriers to help-seeking by community professionals even after the ED Programme, as they do for young people themselves and their families [15]. In this context, further research is required about whether more broadly focused, and potentially more inviting, support services for young people; planned and publicised with input from young people who have experienced psychosis in order to avoid potentially off-putting aspects of services and the language used to describe them, can help identify people experiencing a first episode of psychosis promptly and influence DUP and pathways to care. The current evaluation of a youth mental health pathway and online youth mental health website in Birmingham UK [40] provides an example of this.

## Conclusion

An early detection programme aimed at staff in non-health service community organisations was well-implemented but ineffective in reducing duration of untreated psychosis or improving pathways to care for people experiencing a first onset of psychosis. Despite recommendations that EIS services should seek to promote access to services through community engagement [41,42], it is unclear how they should do this effectively, and it may not be the best use of limited resources for EIS services. Further research into effective ways to enhance early detection and reduce duration of untreated psychosis is required.

## Additional file

Additional file 1: Table S1. Participant questionnaires pre and post ED workshops: attitudes to psychosis and mental health services. **Table S2.** Participant questionnaires pre and post ED workshops: expected outcomes for someone with first episode psychosis. **Table S3.** Characteristics and experience of new EIS service users during ED Programme year – research interview data. **Appendix 1.** Measures used in research interviews with service users accepted for treatment by CIEIS. **Appendix 2.** Additional quotations from focus groups with stakeholders of the Early Detection Programme.

## Abbreviations

CIEIS: Camden and islington early intervention programme
DUP: Duration of untreated psychosis
ED Programme: Early detection programme
