# Supplementary material for: Evaluation of a community awareness programme to reduce delays in referrals to early intervention services and enhance early detection of psychosis
Source: BMC Psychiatry. 2015 May 2;15:98. doi: 10.1186/s12888-015-0485-y (PMC4424506; doi:10.1186/s12888-015-0485-y)
Supplement: Additional file 1: Table S1. — Participant questionnaires pre and post ED workshops: attitudes to psychosis and mental health services. Table S2. Participant questionnaires pre and post ED workshops: expected outcomes for someone with first episode psychosis. Table S3. Characteristics and experience of new EIS service users during ED Programme year – research interview data. Appendix 1. Measures used in research interviews with service users accepted for treatment by CIEIS. Appendix 2. Additional quotations from focus groups with stakeholders of the Early Detection Programme. [file 12888_2015_485_MOESM1_ESM.docx]

**Evaluation of a community awareness programme to reduce duration of untreated psychosis and enhance early detection of psychosis**

**Additional files for the Data Supplement**

Table DS1: Participant questionnaires pre and post ED workshops: attitudes to psychosis and mental health services

| Measure | n  (paired samples) | Mean score  pre-workshop (sd) | Mean score post-workshop (sd) | Mean difference  (sd) [CI] | Statistical test |
| --- | --- | --- | --- | --- | --- |
| Knowledge about psychosis  *(range: 0-17; high score = more knowledge)* | 294 | 11.3 (2.6) | 13.0 (1.7) | -1.7 (2.5)  [-2, -1.4] | t= -11.3 (df 281)  p<0.001 |
| Attitudes to psychosis  *(range: 8-40; high score = more stigmatising attitude)* | 312 | 20.2 (3.8) | 18.7 (4.2) | 1.6 (3.5)  [1.2, 2] | t = 7.8 (df 308)  p<0.001 |
| Attitudes to mental health services  *(range: 12-60; high score = more positive attitude to services)* | 280 | 43.6 (5.1) | 47.5 (5.6) | -4.2 (5.4)  [-4.9,-3.6] | t = -12.9 (268)  p<0.001 |
| EIS chosen as first referral destination | 251 | 95 (37.8%) | 168 (68.3%) | +30.5% | Chi^2^ = 27.4  p<0.001 |

Table DS2 Participant questionnaires pre and post ED workshops: expected outcomes for someone with first episode psychosis

| Question | n | Response options | Pre workshop  (n=285) | Post workshop  (n=296) | Statistical test |
| --- | --- | --- | --- | --- | --- |
| Expected outcome for someone with psychosis (no treatment) | 285 | Don’t know | 6 (2.1%) | 2 (0.7%) | Related samples marginal homogeneity test  p=0.774 |
|  |  | Problem will disappear and not return | 14 (4.9%) | 21 (7.1%) |  |
|  |  | Problem will disappear but may return | 60 (21.1%) | 60 (20.3%) |  |
|  |  | Problem will improve but not vanish | 14 (4.9%) | 23 (7.8%) |  |
|  |  | Problem will persist | 92 (32.3%) | 93 (31.4%) |  |
|  |  | Problem will get worse | 99 (34.7%) | 97 (32.8%) |  |
| Question | n | Response options | Pre workshop  (n=290) | Post workshop  (n=298) | Statistical test |
| Expected outcome for someone with psychosis (with EIS treatment) | 290 | Don’t know | 8 (2.8%) | 4 (1.3%) | Related samples marginal homogeneity test  p<0.001 |
|  |  | Problem will disappear and not return | 121 (41.7%) | 150 (50.5%) |  |
|  |  | Problem will disappear but may return | 75 (25.9%) | 99 (33.2%) |  |
|  |  | Problem will improve but not vanish | 84 (29.0%) | 42 (14.1%) |  |
|  |  | Problem will persist | 2 (0.7%) | 3 (1.0%) |  |
|  |  | Problem will get worse | 0 | 0 |  |

Table DS3: Characteristics and experience of new EIS service users during ED Programme year – research interview data

| **Variable** | | **Whole sample**  **(n=63)** | **Service users accepted via ED routes (n=2)** |
| --- | --- | --- | --- |
| Gender | | Male: n=35 (56%) | Male n=1 (50%) |
| Age (years) | | 23.7 (sd = 4.6) | 20.5 (sd = 2.1) |
| Ethnicity | White British | 22 (35%) |  |
|  | White Other | 11 (18%) |  |
|  | Black ethnic groups | 15 (24%) | 2 (100%) |
|  | Asian ethnic groups | 9 (14%) |  |
|  | Mixed and other ethnic groups | 6 (9%) |  |
| In paid employment | | 9 (14%) | 0 (0%) |
| Number of steps in pathway to EIS | mean (sd) | 3.1 (1.4) | 2 (1.4) |
|  | median | 3 | 2 |
| Service DUP (days) | mean (sd) | 623 (1166)  (n=57) | 427  (n=1) |
|  | median | 152 | 427 |
| Satisfaction with EIS  (CSQ score  total range 8-32*) | mean (sd) | 26.9 (4.2) | 22.5 (2.1) |
| Satisfaction with all mental health services (CSQ score  total range 8-32*) | mean (sd) | 23.9 (5.6)  (n=61) | 14  (n=1) |
| Perception of Coercion (AES score total range 0-5*) | mean (sd) | 2.3 (1.6) | 2.5 (0.7) |
| Hope/anticipated stigma (HAS total range 10-50*) | mean (sd) | 30.5 (7.8)  n=62 | 30 (5.7) |
| Premorbid adjustment – childhood (MPAS-A total range 0-10**) | mean (sd) | 2.2 (2.5) | 1 (1.4) |
| Premorbid adjustment – adolescence (MPAS-A total range 0-13**) | mean (sd) | 3.5 (2.5) | 3.5 (0.7) |
| BPRS suicide item (score 0-7*) | mean (sd) | 2.7  (1.8) |  |
| BPRS hostility item (score 0-7*) | mean (sd) | 3.4  (1.7) |  |
| CAARMS Ultra High Risk Status  (whole sample n=62,  ED routes n=2) | No Psychosis | 3 (4.8%) | 1 (50%) |
|  | UHR (vulnerability) | 2 (3.2%) |  |
|  | UHR (BLIPS) | 2 (3.2%) |  |
|  | UHR (attenuated symptoms) | 13 (21.0%) |  |
|  | Psychosis | 42 (67.7%) | 1 (50%) |
| OPCRIT diagnosis | No DSM IV diagnosis | 7 (11.1%) | 1 (50%) |
|  | Major depressive disorder | 2 (3.2%) |  |
|  | Schizophrenia | 13(20.6%) | 1 (50%) |
|  | Schizophreniform disorder | 14 (22.2%) |  |
|  | Schizoaffective disorder (depressed type) | 9 (14.3%) |  |
|  | Schizoaffective disorder (bipolar type) | 3 (4.8%) |  |
|  | Delusional disorder | 1 (1.6%) |  |
|  | Psychotic disorder (other) | 14 (22.2%) |  |
| Number of people identified asocial contacts (not including mental health staff)  Social Network Scale | At least one | 62 (98%) | 2 (100%) |
|  | At least two | 62 (98%) | 2 (100%) |
|  | At least five | 55 (87%) | 2 (100%) |
|  | At least 10 | 33 (52%) | 1 (50%) |
|  | 12 or more | 19 (30%) | 1 (50%) |

* High score: good **Low score: good

**Appendix 1: Measures used in research interviews with service users accepted for treatment by CIEIS**

Study researchers completed a research interview with consenting CIEIS service users as soon as possible once they had been accepted for treatment by CIEIS. The interview schedule sought information about participants’ socio-demographic characteristics and included the following structured measures:

**Pathways to care (Fisher et al. 2008 [27])** The instrument developed by Fisher and colleagues assesses the number of steps in the pathway to EIS care and the types of agency and professional involved, with reference to a checklist of thirteen categories of helping professional. It can be used to categorise pathways to CIEIS as: no other mental health services involved; via mental health services (non-acute); or via acute services (mental health or criminal justice).

**Client Satisfaction Questionnaire (Attkisson and Zwick 1982 [29]).** The CSQ is an eight-item measure of overall satisfaction with services. It was used twice in our study: to rate respondents’ satisfaction with CIEIS; and to rate their satisfaction with all mental health services received. Respondents’ agreement with each statement is rated on a fourpoint likert scale, yielding a total scale scorefrom 8-32.

**Admission Experience Survey -Perception of Coercion Subscale (Gardner et al. 1993 [30])** TheAdmission Experience survey is a 16-item measure of service users’ experience of first contact with mental health services. We used items 1, 4, 7, 14 and 15 from this scale, which constitute the perception of coercion subscale. Respondents agree or disagree with each statement, creating a total score from 0-5.

**BPRS suicide and hostility items (Overall and Gorham 1962 [32])** We used two items from the 24-item BPRS measure of overall psychiatric symptoms. These use structured interview questions to assess self-harm/suicide and hostility/violence towards others on a seven-point scale.

**Nottingham Onset Schedule (DUP) (Singh et al. 2004 [28])** The Nottingham Onset Schedule provides a structured interview process to elicit the date of a respondent’s first psychotic symptom (rating 4 or above on a PANSS assessment) and the date at which effective treatment commenced (adherence to anti-psychotic medication). Rather than the date of treatment starting however, we used the schedule to calculate “service DUP”: ie the date from first psychotic symptom to the date of first contact with CIEIS. This was considered the more relevant measure of access to EIS services.

**Hope/anticipated stigma (unpub.)** A measure was adapted from published tools, including the Illness Perception Questionnaire for Schizophrenia (Lobban et al.). Respondents are asked to rate their agreement with ten statements about their views about their own mental health problem and others’ reaction to it, and their expectations for the future, on a five-point likert scale, yielding an overall score from 10-50, assessing hopefulness and anticipated stigma

Lobban,F. Barrowclough,C. Jones,S. **Assessing cognitive representations of mental health problems 1: The Illness Perception Questionnaire for Schizophrenia** *British Journal of Clinical Psychology* 2005, 44: 147-162

**Social Network Schedule (Dunn et al. 1990 [33])** The Social Network Schedule uses prompting questions to ask respondents to list all their social contacts. The type of relationship, location and frequency of meeting with each contact is then recorded, as well as whether the respondent considers each contact to be a friend or not. We used this measure to assess the number of people (excluding mental health staff) with whom participants were in contact at the time of the research interview.

**Pre-morbid Adjustment Scale (Gupta et al. 1995 [34])** The Premorbid Adjustment Scale developed by Gupta and colleagues asks respondents about their social functioning in childhood (age 6-12) and adolescence (age 13-21). The childhood subscale asks respondents to rate their functioning with reference to withdrawal, peer relationships and interests, yielding a total score from 0-10 (low score = good functioning). The adolescence subscale covers these items and also sexual relationships, yielding a total score of 0-13 (low score = good functioning).

**CAARMS Ultra High Risk categorisation (Yung et al. 2006 [31])** The Comprehensive Assessment of At Risk Mental States developed by Yung and colleagues provides a structured assessment of psychotic and prodromal symptoms and can be used to categorise respondents as: not psychotic; ultra high risk (sub-categorised as vulnerable to psychosis, experiencing “blips” – brief, limited intermittent psychotic symptoms – or attenuated psychotic symptoms; or psychotic.

**OPCRIT diagnosis (McGuffin et al. 1991 [35])** Following the research interview, the study researcher used the information elicited from respondents to complete a 90-item checklist in the OPCRIT computer programme, which enabled generation by OPCRIT of a diagnosis for the respondent, relating to DSM diagnostic criteria.

**Appendix 2: Additional quotations from focus groups with stakeholders of the Early Detection Programme**

**WHAT WERE IMPEDIMENTS TO THE SUCCESS OF THE EARLY DETECTION PROGRAMME?**

**Confusion about the EIS service and referrals**

“is it for young people at risk of psychosis, or is it actually something much broader, which is about young people that are developing risk factors or, you know, engaging in behaviours, smoking large quantities of cannabis, for example. They may or may not be at risk of developing psychosis, but certainly they are more an at risk group. So I don’t know”. Community Manager

**Stigma and fear**

“there’s certainly a hesitation to refer from the youth team that I went to because they didn’t want to stigmatise their clients who they weren’t even sure necessarily was developing a psychosis, they didn’t want to stigmatise him with a referral to mental health”. EIS staff

“that’s the sort of main point. It’s about sort of education and stigma and stuff because in a way if you could change that the sort of people would access services almost naturally and organically”. EIS staff

“often people actually don’t talk about it because it’s this shame or some kind of weakness”. Community staff

**Service user and family factors**

UF Mine totally self-isolates. That’s what she does.

UF2 That’s the thing, isn’t it, people withdraw.

UF That’s the thing, that’s what they do. They withdraw into their own world.

UF3 Yes, that’s what my son did as well. He just withdrew

“But, it’s all the people that aren’t tapped into any organisation, that still aren’t tapped into any organisation, they’re at home with their parents in their bedroom and the parents are probably worried or thinking are they going to grow out of this at some point, is this kind of adolescent behaviour or, you know… and, they’re not really sure what to do and I’m not sure that we’re reaching those people”. EIS staff

“when it happened to me it sort of... it was all normal... I didn’t know anything was wrong with me and sort of... and then I was sectioned”. Service user

**WHAT ELSE CAN WE TRY?**

**Tackling barriers to accessing support**

“So that’s a kind of developing the language and a framework to understand things by”. Manager (JO).

“It has to be out there in the public domain so that people feel comfortable about it”. Carer

Carers often described feeling ignorant and helpless when their loved one experienced difficulties.

“We need to be educated about it, don’t we? I felt so incredibly ignorant”. Carer

**Who to target**

“I think it has got to be the general public and particularly sort of young people that are getting the information first hand”. Community manager

**Reaching people**

“what do you mean Early Intervention Service, so it might be… Certainly from our service perspective, the name creates a problem and a barrier”. Community Manager
